# Injury Prevention Strategies in Female Football Players: Addressing Sex-Specific Risks

**DOI:** 10.3390/sports13020039

**Published:** 2025-02-04

**Authors:** George John, Ameen AlNadwi, Tiffany Georges Abi Antoun, Ildus I. Ahmetov

**Affiliations:** 1 Transform Specialist Medical Centre, Dubai 119190, United Arab Emirates; 2 Laboratory of Genetics of Aging and Longevity, Kazan State Medical University, 420012 Kazan, Russia; 3 Sports Genetics Laboratory, St. Petersburg Research Institute of Physical Culture, 191040 St. Petersburg, Russia; 4 Research Institute for Sport and Exercise Sciences, Liverpool John Moores University, Liverpool L3 5AF, UK

**Keywords:** female athletes, soccer, FIFA 11+, injuries, ACL, menstrual cycle

## Abstract

There has been rapid growth in women’s football worldwide; however, research on injury prevention strategies and physiological considerations specific to female athletes remains insufficient. Women’s football has experienced an increase in injury prevalence, despite being underrepresented in sports literature, with less than 25% of research focused on this demographic. The incidence of injuries, particularly among young elite female footballers, is notably high, impacting long-term health outcomes such as osteoarthritis and contributing to shorter playing careers. Certain injuries, such as anterior cruciate ligament (ACL) ruptures, occur at significantly higher rates in women compared to men, underscoring sex-specific risk factors that must be addressed in injury prevention programs (IPPs). This narrative review aims to evaluate the effectiveness of IPPs tailored for female football players and to address their heightened susceptibility to injuries compared to males. Research studies and review articles were identified using a literature search of the PubMed, SportDiscus, and Google Scholar databases from 1990 to December 2024. Biological factors, including hormonal influences—such as increased ACL laxity during the menstrual cycle—and musculoskeletal differences, such as muscle strength imbalances, reduced joint stability, and hip weakness, significantly contribute to this increased risk. Despite the existence of injury prevention protocols like FIFA 11+, their consistent application and adaptation to meet the unique needs of female footballers remain limited. In addition to physical injuries, mental health is a critical concern. Female football players exhibit higher rates of depression and anxiety compared to their male counterparts, influenced by factors such as injury-related stress and menstrual cycle variations. In conclusion, the growing participation of women in football highlights the urgent need for research and implementation of injury prevention strategies specifically tailored to female players.

## 1. Introduction

### 1.1. A Historical Perspective on Women’s Football

The history of women’s football in England, Europe, and the USA is both controversial and community-driven. The first international women’s match occurred on 9 May 1881, with Scotland defeating England 3–0 [1]. On 23 March 1895, the British Ladies Football Club organized the first recorded game in England, playing over 100 matches across Britain [1]. Between 1917 and 1921, women’s football gained popularity in Britain due to changes in women’s roles and social movements. However, on 5 December 1921, the Football Association (FA) banned women’s teams from League and Association grounds, claiming football was too vigorous for women and citing financial concerns in charitable games [1]. This ban persisted and was periodically enforced, notably in 1946 and 1949.

Women’s football struggled for acceptance until the men’s 1966 World Cup victory reignited interest. By 1969, the FA affiliated with the World Federation of Advertisers, and media coverage of women’s football began increasing by 1989. The success of the 1990 men’s FIFA World Cup shifted the style of play to emphasize skill and finesse, influencing the women’s game. In 1989, the FA started providing coaching and support for women’s football [2].

### 1.2. The Global Rise of Women’s Football

Women’s football has seen rapid global growth, with FIFA reporting 30 million female football players in 2014. By 2018–2019, over 28,000 women participated in the National Collegiate Athletics Association (NCAA) women’s football, making it the second leading sport for collegiate women in the United States, just behind outdoor track and field [3].

According to a recent study by the Sports Consultancy, the global women’s sports market is projected to nearly triple by 2030, with over 13 million women playing organized football worldwide [4]. In addition, there are currently more than 30 elite women football national leagues well established in different countries, mainly in European countries [5]. According to the Women’s Football: Member Associations Survey Report 2023 [6], there were 16.6 million women and girls playing organized football in 2023, representing a 24% increase since 2019. Over the same period, significant growth in participation was observed in countries such as Brazil (a 5500% increase since 2019), Mexico (2900%), and England (2720%) [6]. Despite such growth of women in football, support during and post-retirement remains limited, leading to higher injury rates and mental health issues compared to men [7].

### 1.3. Challenges Faced by Women in Football

The prevalence of injuries is increasing in women’s football. Despite football being the most abundantly studied sport in scientific literature, less than 25% is conducted on women’s football [7]. The injury incidence rate in women’s football is notably high, particularly among young elite female footballers [8]. For instance, a study of 498 girls participating in the Danish Football Association series found that players aged 15–18 sustained a total of 424 injuries, highlighting the prevalence of injuries in this age group [9]. Such a high injury burden can have long-term consequences, as evidenced by research showing that 30% of former Polish female football players ended their careers due to prolonged injury treatment [10]. It is also evident that frequent injuries in females increases the risk of osteoarthritis (OA) [11]. According to Le Gall et al. [7], 83.4% of injuries in young elite players (ages 15–19) were located at the lower extremity. Additionally, the relative risk of anterior cruciate ligament (ACL) injuries occurring in women is substantially greater than men [12,13].

Sports-related injuries are an on-going public health concern in several countries, with approximately 30% of injuries among children and adolescents stemming from sports-related incidents and regular sports participation [11]. To address this issue, Injury Prevention Programs (IPPs) have been developed and implemented to mitigate the frequency and severity of these injuries. IPPs are an inherent part of training in recreational and professional sports training. They not only enhance performance, but also prevent injuries, which could influence coaches and athletes to prioritize injury prevention in their daily routines to mitigate the increasing prevalence of injuries. IPPs typically focus on three main areas for injury prevention: training strategies, rule and policy modifications, and equipment recommendations [14], aiming to tailor interventions to specific sports and injury mechanisms.

However, despite their implementation, IPPs have frequently used a broad approach, potentially overlooking important aspects like the player’s sex. Research indicates that men and women respond to IPPs differently, which emphasizes the need for tailored approaches that consider sex-specific factors to effectively prevent injuries in women [15]. Mendonca et al. [11] argues that IPPs should be evidence-based, widely adopted, properly implemented, sustainable, and continuously evaluated to optimize effectiveness, adherence and safety for all athletes.

Based on current research, there appear to be potential problems in regard to women in football; IPPs are not as effective in preventing injury compared to IPPs for men [16]. Scientifically, women’s physical bodies and physiology are different compared to men; thus, their injury risk profile is different. The incidence of ACL ruptures in women is at least double compared to men [17] but the lack of research conducted on the efficacy of IPPs does not provide incentive toward tailoring specific IPPs towards women and their sex-specific needs [18]. Regardless of IPPs placed by professional sporting organizations, such as FIFA 11+, Prevent Injury and Enhance Performance programme (PEP), overall injuries among professional women football players were reduced by only 22–27% compared to 27–40% for men [19]. The issue present is not related to adherence of IPPs, but rather to how men and women’s physical bodies respond differently to such programs. Another issue presented among the research is the implementation of IPPs in professional sporting contexts. Although IPPs reduced the incidence of ACL ruptures in women’s football by 45% [20], a lack of implementation and consistency within IPPs can reduce their ‘real-world’ effect to 13% [21].

This literature review aims to achieve three main objectives. Firstly, it seeks to assess the suitability of IPPs for female football players, while also evaluating their effectiveness. Secondly, it will examine the higher risk of injury among women compared to men in football, focusing on specific injuries that female players are prone to. These include, but are not limited to, ACL injuries, muscle injuries, and concussions, which severely affect female athletes. Finally, the review will identify the current limitations of existing IPPs and recommend improvements aimed at enhancing injury prevention and overall health of female football players.

Furthermore, the purpose of this review extends to understanding the biological factors contributing to women’s higher injury susceptibility in football. It also explores differences in how women respond to IPPs compared to men, emphasizing the need for tailored approaches in professional football. For example, IPPs can be tailored toward women in football by considering factors such as menstrual cycle, genetic predisposition towards injury, and effective implementation and follow-up strategies. Since women’s football is one of the fastest growing sports [22], adequate and reliable research is required to establish proactive and preventative strategies toward injury prevention, as well as to gain a deeper understanding of women’s physiological needs in sports.

## 2. Literature Search

To identify relevant research studies and review articles, a comprehensive literature search was conducted across the PubMed, SportDiscus, and Google Scholar databases, covering the period from 1990 to December 2024. The search strategy involved screening article titles, abstracts, and keywords for specific terms using Boolean operators (“OR” and “AND”) and truncation (“*”) to maximize the search scope. To enhance the comprehensiveness of the search, MESH terms were employed in PubMed, for example, “Soccer”, “Football”, “Injuries”, “Female Athletes”, “Anterior Cruciate Ligament”. These were combined with free-text terms to maximize the identification of relevant studies. Overall, the key search terms included, but were not limited to, Soccer, Football, Female, Women, Girls, Adolescents, Injury, Injury Prevention Strategies, ACL, FIFA 11+, Menstrual Cycle, Individual Responses, Polymorphism, and Genetic. These terms were combined in various configurations to ensure a broad yet focused capture of relevant literature, for example, (“Soccer” OR “Football”) AND (“Female” OR “Women” OR “Girls”); (“Injury” OR “ACL” OR “Injury Prevention Strategies”) AND (“Menstrual Cycle” OR “Polymorphism”).

Inclusion Criteria:

Publications were included if they:

1. Contained relevant data on the physiology, injury profiles, or injury prevention strategies specific to female football players.
2. Focused on topics of genetic, physiological, or training-related factors associated with female football performance or injuries.
3. Were written in English.

Exclusion Criteria:

Studies were excluded if they:

1. Did not address female football players or their specific physiological/injury characteristics.
2. Were not peer-reviewed journal articles (e.g., conference abstracts, non-scientific reports).
3. Were not written in English.

Search range and scope: Studies were not excluded based on the competitive standard of the football players (e.g., elite vs. recreational) or the geographic location of the study origin. This approach was adopted to ensure a diverse and inclusive overview of the literature.

Additional efforts: Backward citation tracking (the snowball search method) was employed by reviewing the reference lists of relevant studies (especially systematic reviews) to identify any additional articles that met the inclusion criteria. This iterative process helped ensure comprehensive coverage of the topic.

## 3. Literature Review

### 3.1. Sex Differences in Injury Risk

**Figure 1:**
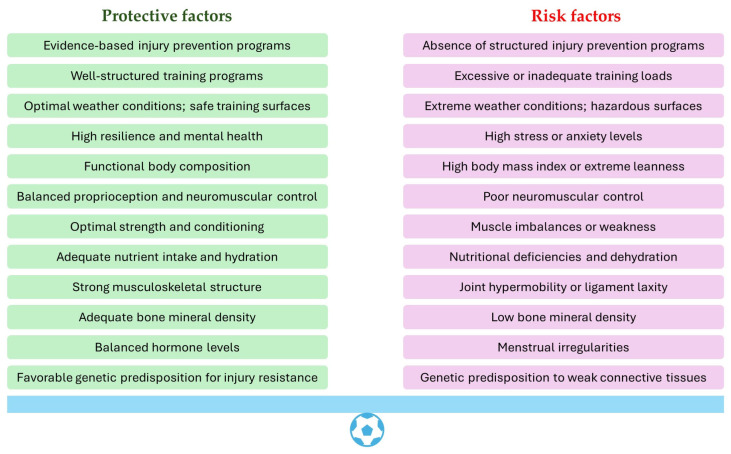
Risk and protective factors for injuries in female football players.

Generally speaking, women are three times more likely to get injured compared to males, whether that is in the workplace or in a sporting environment, as women struggle with more musculoskeletal issues due to undesirable movement patterns [23]. While previous research on sport-specific injury patterns has primarily focused on men, the increasing participation of women in sports reveals gender-specific injury trends, suggesting that women may be at a greater risk for certain injuries [24]. Figure 1 highlights some of the key risk and protective factors that can influence injury rates in female football players. In football, there seems to be different physiological demands placed for women compared to men, whether that is considering reproductive health, recovery time or psychological health. It is crucial to apply existing knowledge on the risk of injury to address the specific needs and experiences of female athletes [3].

An elite women’s football team can expect approximately 35 time-loss injuries per season. This is illustrated in a UEFA Women’s Elite Club Injury study, where 596 players from 15 elite women’s teams in Europe were studied during the 2018/2019 to 2021/2022 season [25]. However, it should be noted that the absolute count (e.g., 35 time-loss injuries per season) may vary depending on the squad size. The results also showed that 1527 injuries were recorded in 463 players with an injury incidence of 6.7 injuries per 1000 h of play [25]. Thigh muscle injuries accounted for 12% of all injuries, followed closely by quadriceps injuries at 11%. Above all, ACL injuries posed the highest burden. Additionally, while concussions were the most frequent head injury, they accounted for only 3% of all injuries. These were primarily caused by being hit by a ball (34%) or heading the ball (21%), highlighting that head and facial injuries constitute a small proportion of the overall injury profile. This establishes the prominence of muscle injuries in elite women’s football, with hamstring and quadriceps injuries being prevalent [25].

### 3.2. The Female ACL: Anatomy, Physiology, and Vulnerabilities

An anterior cruciate ligament injury is the overstretching or tearing of the anterior ACL in the knee. An ACL injury can occur when an individual is struck on the side of the knee, such as during a football tackle, when the knee joint is overextended, or as a result of quick and sudden movements while running or landing from a jump. Depending on the severity of your ACL injury, treatment may include rest and rehabilitation exercise to help with regaining strength and stability, or surgery to replace the torn ligament followed by rehabilitation [26].

Although the general population faces a relatively low risk of ACL injury, elite football players, both men and women, are highly susceptible to ACL injuries, which are considered prominent in the sport [13]. While football is viewed as a relatively safe sport for males, the increasing participation of women in football has led to a rise in reported ACL ruptures [27]. Based on the current literature, women in professional football are 2–8 times more likely to suffer ACL injuries compared to male players [28]. With approximately 10% of the 260 million active football players being female [29], in-depth research is required to determine what factors contribute to the increase in ACL ruptures. Ireland [28] suggests that there are a number of factors contributing to the discrepancy in ACL injuries in females, including hormonal and anatomical differences, neuromuscular adaptations, and core stability.

Research consistently indicates that hormonal fluctuations during the menstrual cycle influence the risk of ACL injuries in female athletes, although findings remain partially inconsistent. Several studies highlight that ACL injury risk peaks during the late follicular and ovulatory phases, coinciding with elevated estrogen levels. For instance, Shultz et al. [30] demonstrated that knee laxity increases as estradiol levels rise, while Martin et al. [31] observed a 47% and 32% higher injury risk during the late follicular phase compared to the follicular and luteal phases, respectively.

Consequently, Ireland’s [28] research aims to increase public awareness regarding the increased risk of ACL injury for women. Sex hormones significantly influence ACL rupture rates in women. During certain phases of the menstrual cycle, levels of sex hormones, such as estrogen and progesterone, can fluctuate dramatically, with concentrations potentially doubling or halving within a 24 h period. Along with this, they go through an assortment of physiological and psychological changes that influence athletic performance and overall well-being [32]. Wojtys et al. [33] found that more ACL injuries are reported during the ovulatory phase of the menstrual cycle compared to the follicular phase. Another study conducted by Wojtys et al. [34] measured estrogen, progesterone, and luteinizing hormone metabolites in the urine to determine whether there is an association between the menstrual cycle phases and ACL injuries in female athletes. The authors concluded that ACL injuries are more likely to occur during the ovulation phase of the menstrual cycle, possibly due to the increase in estrogen cycling through the body, which has physiological effects on soft tissues; however, it should be noted that not all studies report a peak around ovulation [34].

Additionally, high levels of estrogen have been linked to structural changes in the ACL, affecting its composition and mechanical strength. This hormonal influence may reduce ACL strength by downregulating fibroblasts, thus increasing the risk of injury to the ligament. Moreover, heightened estrogen levels contribute to increased joint laxity around the knee, further weakening joint stability [35]. The laxity around the knee decreases joint stability, therefore increasing the likelihood of undesirable movement patterns and injury [36]. Such findings emphasize the interplay between hormonal fluctuations and ligamentous properties, though conflicting results highlight the need for larger, multi-cohort studies to confirm these associations.

Apart from female sex hormones, testosterone levels may also influence injury risk in football players through their effects on muscle strength, recovery, and ligament properties, although the relationship is complex and varies by sex. Studies indicate that testosterone levels are positively associated with sprinting ability in female athletes [37] and may increase throughout the season in female football players [38]. In a pilot study, it has been shown that the female ACL is an androgen-responsive tissue, with testosterone and the free androgen index being correlated with ACL stiffness near ovulation [39]. However, the androgen-responsiveness of the female ACL requires further investigation to elucidate its contribution to injury prevention.

These findings collectively suggest that hormonal fluctuations, particularly those involving estrogen and testosterone, interact with biomechanical and neuromuscular factors to influence ACL injury risk in female athletes. Future research should aim to integrate hormonal, mechanical, and other data to develop personalized, phase-specific injury prevention programs for female football players.

Furthermore, anatomical differences between the sexes are perhaps another important factor to discuss regarding the increased risk of ACL injury in females. A common explanation as to why physical differences predispose women to ACL injury is due to women having wider hips [40] which creates biomechanical differences regarding knee alignment. Thus, women are more likely to suffer from dynamic knee valgus, which has been linked to ACL injury risk [41]. Cheung et al. [42] highlighted key anatomical differences, such as tibial anatomy, femoral notch morphology, and ACL size, as possible explanations to the increased incidence of ACL injury in females. According to tibial anatomy differences, women tend to have an increased posterior tibial slope (PTS) compared to men [43]. The PTS is an anatomical structure that is directly associated with anterior tibial translation, knee stability, and internal rotation of the knee joint during weight-bearing conditions [44]. Due to the greater degree of the PTS, women are more likely to strain their ACL due to increased internal rotation of the knee during pivotal landing [45].

Another anatomical structure that can influence injury risk is the width and shape of the femoral notch; this is determined by the size and orientation of the ACL [28]. Typically, women have a narrower and smaller femoral notch compared to men [42]. Based on the literature, a narrow femoral notch is an independent risk factor for ACL injury [46]; one possible explanation for this is that a smaller notch leads to impingement of the ACL on the lateral aspect, therefore resulting in increased strain on the ligament [42]. Additionally, some research has suggested that the size and morphology of the female ACL is smaller when compared to males, and a smaller ACL is more likely to rupture compared to larger ligaments [42]. According to Lipps et al. [45], the difference between structural properties of the ACL led the authors to examine how peak ACL forces are affected during a simulated pivot landing. Their research illustrated that female cadaveric knees showed 95% increased strain during the simulation compared to males [45]. Other theories suggest that ACL and femoral notch sizes are related and have important implications as to why females are at an increased risk of rupture [47].

The current literature suggests that female anatomy plays a big role in heightening their risk of injury; thus, extensive research is needed to understand how these differences can be taken into consideration when providing women with injury prevention programs.

Moreover, neuromuscular control is directly related to dynamic joint stability; it is the unconscious trained response of the muscles that is responsible for contraction, coordination, stabilization, postural control, and balance [48]. Neuromuscular control is crucial when it comes to sport since it protects the body during dynamic movement patterns, such as landing, as it ensures biomechanical stability [48]. Altered neuromuscular coordination is another common cause of knee injury, especially for the ACL [35]. Females differ in muscular control, activation and movement patterns compared to males when performing in sports; thus, these differences in neuromuscular adaptations can be another explanation towards their increased injury risk in the lower extremity [48]. In addition, it is reported that women are less effective in stiffening their knee; therefore, without maximal contraction of the knee musculature, the joint capsule is more prone to anterior tibial translation, which is the primary movement pattern that results in ACL sprain or complete rupture [49].

To counteract anterior tibial translation, the hamstring muscles play a vital role in acting as a posterior force to the tibial, thus supporting the ACL during landing and dynamic movement patterns [35]. When comparing musculoskeletal changes during peak developmental phases for girls and boys, the literature suggests that females display weaker hamstring strength relative to their quadriceps muscle during puberty compared to males [35]. Moreover, males show significant increases in peak concentric and isometric hamstring muscle torque during puberty, while females display no significant increases [35]. This comparison in hamstring muscle strength is another neuromuscular adaptation that occurs in females that predisposes them to ACL injury [35]. Wojtys et al. [50] demonstrated that females take longer to produce maximal hamstring torque during isokinetic testing compared to males. So, in response to anterior tibial translation, females recruit the quadriceps muscle instead of the hamstrings for knee stabilization [50]. Huston and Wojtys [49] supported this by examining neuromuscular performance characteristics in elite female athletes and reporting that only 28% of female athletes preferred to recruit their hamstring muscle as an initial response to anterior tibial translation compared with 45% of male athletes.

More recent research using advanced techniques, such as 3D motion analysis and electromyography, has refined these findings by providing greater insights into sex-specific neuromuscular control strategies. For instance, in a study of 205 female athletes in high-risk team sports (football, basketball, and volleyball) using three-dimensional kinematics (joint angles), it was shown that knee motion and knee loading during a landing task can predict ACL injury risk in female athletes [51]. Electromyography studies have also identified specific EMG patterns associated with ACL injury risk in female team sport athletes [52]. These contemporary analyses validate and expand upon earlier work, reinforcing the importance of targeting neuromuscular training to address these deficits in female athletes.

### 3.3. Core Stability as a Risk Factor for Injuries in Female Football Players

Moreover, up to 70% of ACL injuries are caused by non-contact mechanisms, which indicates that muskuloskeletal factors, such as muscle strength/endurance or joint stability, greatly influence injury risk [53]. According to De Blaiser et al. [54], impaired core stability is a risk factor for lower extremely overuse injuries. Researchers conducted a prospective cohort study that demonstrated 24% of their participants suffered from an overuse injury that was primarily due to decreased abdominal core muscle endurance, along with dynamic postural control and weak hip flexion/extension isometric strength. Based on the recent literature, it has been determined that distal function of the lower extremities is influenced by proximal control [28].

One concept linked to this idea is core stability, which encompasses multiple dimensions, including motor control, endurance, and trunk stiffness. Muscles surrounding the abdomen play a crucial role in stabilizing the lumbopelvic region and the spine [55]. The relationship between a strong core and the stability of the lower extremity is defined by an optimal kinetic chain, reducing forces on the lower limbs and enhancing stability during dynamic movements. Without sufficient core strength, even strong lower extremity muscles may result in inefficient movements due to the lack of force generated in the core, leading to inadequate energy transfer [56]. This creates an unstable foundation for safe and controlled movement patterns [57].

Core stability is assessed through a variety of methods in the literature, including plank endurance tests, balance assessments, and measures of trunk stiffness or motor control. Leetun et al. [58] found that males generally demonstrate greater core stability than females, and their study indicated that athletes with lower core stability measures were more likely to experience injuries. Women may have relatively less stable cores compared to men, potentially due to anatomical differences such as a wider pelvis, which alters the angle of core muscle attachment and affects force generation [59].

Along with hip muscle weakness and lack of proprioception/balance deficits in the lower extremity, lack of core strength is another risk factor that has been identified to increase the risk of ACL injury for females [60]. Zazulak et al. [61] supported this notion by demonstrating that impaired core proprioception is a strong predictor of knee injury risk, with 56% specificity for female athletes and not male athletes [61]. Prior research indicates that core stability and strength is a strong indicator of athletic success and sport-specific movements [59]; however, lack of strength and stability in the lumbopelvic region produces inefficient movements and an abnormal agonist/antagonist relationship, which is a common risk factor that could lead to injury [28].

Therefore, this relationship between core stability and increased injury risk should provide incentive towards developing effective core stabilization programs as well as implementing functional core training for female athletes. These programs should target women since they are already predisposed to a weak core due to anatomical differences when compared to male counterparts.

### 3.4. The Impact of Mental Health on Female Football Players

Based on existing literature, the primary focus has largely centered on physical and mental resources relevant to the male experience in sports. In men’s football, recent study has shown the that prevalence of mental health symptoms ranges from 10% for distress to 19% for adverse alcohol use, and 26% for anxiety and depression [62]. These statistics differ markedly when compared to women in football. Although research on women in football is limited, data indicate a higher prevalence of mental health symptoms, with rates ranging from 13% for depression to 44% for alcohol misuse and 63% for sport-related distress [63]. These findings suggest that, despite limited research, female football players experience a higher burden of mental health challenges than their male counterparts. This disparity is influenced by factors such as injuries, hormonal fluctuations related to the menstrual cycle, and inadequate support during and after their football careers.

To address these challenges, this study examines the factors affecting the mental health of women in football and evaluates whether current interventions are sufficient or require improvement. Notably, women’s health has been historically underrepresented in sports science research, with only 8% of studies focusing on conditions unique to women, up from 6% in 2020 [40]. Despite this underrepresentation, understanding the menstrual cycle and other physiological differences is critical for optimizing training and ensuring peak athletic performance.

It is well established that the menstrual cycle significantly impacts female athletes’ physical and mental performance, influencing muscle function, energy levels, endurance, and mental well-being. A 2021 study on female football players revealed that all participants reported negative effects from their menstrual cycle, with 94% experiencing reduced power, 87% increased fatigue, and 67% impaired confidence and focus [64]. Despite evidence linking Common Mental Disorders (CMDs) and their symptoms to diminished performance, reduced quality of life, and potential early exit from sport, research on mental health in women’s football remains scarce [64]. CMDs, which include depressive disorders, anxiety disorders, eating disorders, and sleep disturbances, manifest as distress, burnout, depression, and anxiety [63]. Limited data show that the prevalence of depression symptoms in female football players ranges from 13% to 39.7%, and anxiety symptoms range from 1.1% to 7.3% [64].

Furthermore, studies consistently demonstrate that female athletes exhibit higher rates of CMD symptoms than their male counterparts, making them more susceptible to mental health challenges and associated performance consequences. For example, one study examining depression and anxiety symptoms in elite male and female footballers found that 13% of female first-league (FL) players reported depressive symptoms compared to 6.6% of male FL players [64]. Additionally, male FL players reported significantly lower anxiety levels than female FL players. A separate study of 290 German female football players revealed that, among FL athletes, 16.6% experienced mild to moderate depression symptoms and 14.1% experienced severe symptoms [65]. For generalized anxiety disorder, 6.9% reported moderate symptoms and 1.4% severe symptoms. Interestingly, second-league (SL) players reported even higher rates of mental health symptoms; 25.4% experienced mild depression symptoms and 20.6% severe symptoms, while 9.2% experienced mild anxiety symptoms and 4.6% severe symptoms. These findings highlight the heightened mental health challenges faced by SL female footballers compared to their FL counterparts. Overall, while the available research underscores the significant mental health challenges faced by female football players, further investigation is crucial to fully understand these issues and develop effective, evidence-based interventions tailored to the needs of women in football. Furthermore, given that the existing IPPs do not adequately address mental health disorders, it is crucial to emphasize the need for a more comprehensive approach that integrates mental well-being, ensuring that female football players receive support for both physical and mental health challenges.

### 3.5. Adapting Injury Prevention Programs (IPPs) for Female Footballers

In professional sporting environments, athletes are participating in IPPs consistently in order to reduce the risk of acute or overuse injuries. With more females participating in sport each day, research indicates specific sex differences in injury risk and prevention [15]. Thus, the existing literature on injury risk for females should be used to tailor IPPs based on the female experience. Sex-specific IPPs should be implemented by sporting organizations to ensure that both males and females in sport are optimizing their protection against injury [16].

Salam et al. [66] gathered existing literature and concluded that IPPs were effective in decreasing the incidence of injuries in general. IPPs seem to be effective and reduce risk of injury by at least 40% in youths and adults; these programs include strength training, proprioception, balance and psychological strategies to prevent injury [16]. Much of the research is conducted on preventing sport-specific injuries, especially in football. The FIFA 11 and FIFA 11+ programs are IPPs developed by FIFA that combine neuromuscular training, technique and balance in order to eliminate injury. These programs reduced the overall injury incidence by 34%, lower limb injuries by 29% [67], and head and neck injuries by 40% [68].

Furthermore, Soligard et al. [69] found that the FIFA 11+ program reduced injury risk by 32% in female youth football players. Although much of the data provided on IPPs is on male athletes, and many programs do not consider anatomical differences between males and females, recent research on injury prevention for females, specifically on ACL injuries, has been conducted [70]. IPPs have been demonstrated to be highly efficacious in protecting young athletes from ACL and other lower-extremity injuries. However, the effectiveness of these programs in practice has been limited due to poor adherence among coaches of organized sport teams [70].

Several studies highlighted the efficacy and cost-effectiveness of the FIFA 11+ Kids program for young football players. Ramos et al. [71] reviewed 11 studies involving over 10,000 participants aged 7–14 years, primarily soccer athletes, and found a 50% reduction in overall injuries and nearly 60% in severe injuries, with a dose–response relationship for weekly sessions. The program also improved physical performance (e.g., balance, jump tests, soccer skills) and attention (13–18% improvement in ASESC scores). For instance, in the study by Rössler et al. [72] involving both boys and girls playing football in Switzerland, a 51% reduction in injury-related healthcare costs was demonstrated, with nationwide annual savings of CHF 1.48 million estimated with program implementation. These studies support the widespread adoption of the FIFA 11+ Kids program to enhance injury prevention, performance, and cost efficiency in youth football. However, evidence supporting the effectiveness of the FIFA 11+ program in females is limited, and further research is needed [73].

Exercise-based IPPs aim to improve whole-body biomechanics through exercises focusing on strength, balance, mobility, agility, plyometrics and running. Some common IPP’s include FIFA 11+, PEP, and Footy First. FIFA 11+, developed by FIFA’s medical research center, involves 10–15 min of exercises designed to reduce risk of injuries, specifically ACL injuries. Footy First also aims to reduce leg injuries in football [74], emphasizing core stabilization, eccentric training of thigh muscles, proprioceptive training, dynamic stabilization and plyometrics with straight leg alignment.

A recent systematic review identified that for female football players, multiple-component IPP programs have reduced overall injury rates by 27% and ACL injury rates by 45% [18]. However, the effectiveness varies between cohorts. For example, the PEP program significantly reduced ACL injury rates in female players yet showed no significant impact on collegiate-level players. This inconsistency is often due to improper or unsustained implementation of the prevention programs.

The charity ‘Power Up To Play’ (PUTP) is the first of its kind in the UK, aiming to reduce knee injuries in children playing sports through a standardized evidence-based warm-up. PUTP addresses barriers to IPP implementation by providing an accessible framework for players and coaches [75]. According to PUTP, youth knee injuries requiring surgery have increased 29-fold in the last 20 years, often keeping children away from sport for 12 months, and this can end up having long-term health consequences.

Future studies should address known competency, organizational and leadership barriers to implementation, and improve monitoring and reporting methods. Despite some reduction in injuries due to IPPs such as FIFA 11+ and PEP, women’s football sees only a less significant reduction in injuries compared to men’s football. More specifically, a recent meta-analysis has shown a 22% reduction for overall injuries in intervention groups compared to control groups for exercise-based programs (11,773 participants) [18]. Furthermore, studies with multiple training components showed a significant 27% reduction [18]. This 22–27% reduction is lower than the previously reported rates of 27–40% for overall injury reduction in male football players using the FIFA 11/FIFA 11+ programs [19]. Interestingly, for ACL injuries, multiple training component studies demonstrated a significant 45% reduction in female football players [18].

Inconsistent implementation obstructs the effectiveness of IPPs. Consequently, understanding and addressing the biological differences and injury risks unique to women is crucial for improving injury prevention strategies. However, research in this area is lacking, which is one of the many limitations that need to be highlighted. In addition, the perception that ACL IPPs require extensive commitment from players and coaches has impeded widespread acceptance and utilization by athletes and teams to implement the training necessary to reduce ACL injury risks.

### 3.6. Personalized Injury Prevention in Female Football: The Role of Genetics

While some athletes exhibit remarkable resistance to injuries, likely due to favorable genetic factors that influence tissue strength, recovery capacity, and neuromuscular coordination, others are genetically more susceptible to injuries. For instance, the genetic contribution to ACL rupture, estimated at around 69%, is significant and indicates a strong familial clustering of the injury [76]. It should be noted, however, that heritability estimates often vary across populations and methods (e.g., twin studies vs. large-scale registries). Recognizing this high genetic risk could enable clinicians to provide more informed advice to athletes with a family history of ACL rupture. As such, the potential for genetic testing to support a personalized injury prevention strategy for female football players presents a promising direction for future research, especially as the sport continues to grow in popularity and intensity.

Genetic variations, such as single-nucleotide polymorphisms (SNPs), have been associated with traits influencing both football performance [77,78,79] and injury susceptibility [80,81,82]. For example, specific SNPs in genes like actinin alpha 3 (*ACTN3*), collagen type I alpha 1 (*COL1A1*), collagen type V alpha 1 (*COL5A1*), and matrix metallopeptidase 3 (*MMP3*) have been linked to muscle fiber composition, collagen structure, and extracellular matrix degradation, all of which can affect an athlete’s risk of injuries such as ACL tears, hamstring strains, or chronic soft-tissue injuries [83,84,85,86,87,88]. Similarly, genetic variants associated with greater strength (e.g., *HIF1A*) have been shown to reduce the risk of hamstring injuries in football players [82,89,90,91]. These genetic markers, along with others, may eventually facilitate the identification of athletes at increased risk for specific injuries, thereby enabling practitioners to implement targeted injury prevention strategies, such as individualized strength and conditioning regimens or optimized training loads. However, due to the relatively small cohorts of professional or semi-professional players, many of these genetic studies remain exploratory.

These associations emphasize the complex interaction between genetic and environmental factors in injury risk, with training, playing conditions, and recovery protocols also playing key roles. While some genetic markers are linked to football injuries and performance [92], the evidence is limited by small sample sizes and lack of replication. Larger, multi-cohort studies involving elite female players are needed to validate these findings and explore new genetic regions using genome-wide association studies (GWASs) [78,93] and meta-analyses [94,95]. This will help identify genetic variants specific to female athletes, whose unique profiles affect performance and injury risk.

Although integrating genetic testing into injury prevention shows promise, current evidence is insufficient to support its widespread use [96]. As sports genomics advances [97,98], collaboration between researchers, clubs, and national teams is necessary to establish a reliable framework. Further research could enable genetic testing to complement traditional methods in talent identification, training, and injury prevention, providing a more individualized approach to supporting female football players’ health and performance [99]. On the other hand, the analysis of gene expression in response to training in football players may provide valuable insights into physical performance potential and metabolic adaptations, aiding in the optimization of training regimens and the management of body composition [100,101], particularly since body composition is one of the factors influencing injury risk [102,103].

Overall, although genetic testing shows promise in enhancing injury prevention strategies, current evidence does not support its widespread clinical application. Collaborative efforts among researchers and practitioners will be crucial in translating emerging findings into practical tools that complement traditional injury prevention methods.

### 3.7. Strengths, Limitations and Practical Applications

The review provides a comprehensive overview of the key injuries faced by female football players, particularly ACL ruptures, which occur at significantly higher rates in women compared to men due to unique biological, physiological, and hormonal factors. It also emphasizes the importance of considering mental health, an area often underrepresented in sports literature, by highlighting the higher prevalence of depression, anxiety, and stress-related disorders among female athletes. The review addresses a timely and relevant topic, as women’s football is growing rapidly worldwide, yet the research on injury prevention and mental health support specific to female players remains insufficient. By taking a multifaceted approach, the review recognizes the importance of addressing both physical injuries and mental health challenges to ensure the overall well-being of female football players.

Although the current literature provides strong evidence regarding the female experience within football, the differences in their injury risk profile, and the effectiveness of IPPs, several limitations were identified during our research. Apart from the insufficient research conducted on women in sports overall, the majority of the data on women’s injuries in football focus on knee injuries, with limited data on the prevalence of other injuries in different body structures, such as ankle or shoulder injuries. This creates a misleading perception of lower injury incidences in regions other than the knee simply due to a lack of reports on different injury types. For example, Crossley et al. [18] carried out a large systematic review and meta-analysis on the safety of football for women; however, the authors stated that overall injury rates were inconsistent. Based on the multiple articles reviewed for their research, upper limb injuries are not typically recorded, leading to underestimated injury incidence reports [15]. Another limitation is the abundance of reviews and meta-analysis conducted on this topic, whereas longitudinal and/or prospective research would provide stronger evidence-based strategies to enhance the sporting experience in football for women.

Moreover, global sporting organizations such as FIFA have implemented IPPs for their athletes, which have proven effective in reducing the incidences of lower extremity and overall injuries [19]. However, what appears to be missing from the research is the dose that is needed to maximize the efficacy, delivery, and outcome of these programs. The data do not clearly report the duration, frequency, adherence or follow-up measures that should accompany IPPs; a clear roadmap for athletes to follow is needed in order to protect them from injury and decrease the likelihood of re-injury. Furthermore, additional methods for managing mental disorders experienced by female football players should also be implemented in IPPs. Since our review indicates that women respond differently to sports and sports-specific injuries based on their physiology and anatomy, it can be assumed that their pre- and post-IPP responses will differ compared to men. There is also a lack of research evaluating the effectiveness of IPPs on injury incidence during training or matches. This is particularly relevant given recent reports indicating that the match injury incidence rate is nearly six times higher than the training injury incidence rate in female football players [104]. Thus, future research should include follow-up and adherence measures within IPPs and identify how these factors should be specific to female athletes.

The review’s findings suggest that injury prevention programs need to be tailored to address the specific physiological characteristics of female athletes, such as hormonal fluctuations and muscle imbalances, which contribute to higher injury rates, particularly ACL injuries. While programs like FIFA 11+ have shown promise, they must be adapted to more effectively meet the unique needs of female football players. Additionally, given the heightened mental health challenges faced by female athletes, it is crucial to integrate psychological support into IPPs. Addressing mental health alongside physical injury prevention can improve overall well-being and athletic performance. The review also advocates for pre-season screening that includes biological measures, such as hormonal profiling and biomechanical assessments, to identify individual risk factors and allow for more targeted and effective interventions. To better address the needs of female football players, future research should encourage global sports organizations to expand their data collection efforts, ensuring that a wider range of injuries and mental health symptoms are accurately reported. Lastly, the review emphasizes the importance of ongoing evaluation of IPP effectiveness. Clear guidelines on the frequency, duration, and adherence to these programs are necessary, along with regular follow-up assessments, to ensure that they remain effective and continue to meet the evolving needs of female athletes.

## 4. Conclusions

Female footballers are at an increased risk of physical and mental health challenges compared to men. This is mostly due to their physiology, behavior, inadequate IPPs and insufficient resources. Our literature search indicates that female athletes are more prone to injury, depression, anxiety, and other health issues, heightened by poor resources and support. In addition, these adverse effects are likely to decrease performance, impact quality of life and potentially cause an exit from sport. Effective interventions must consider these factors to ensure the well-being and performance of women in football. The insufficient research on injuries in female football can, in part, be addressed by combining data from all available team sports [105]. Another potential suggestion to address the existing issues surrounding women’s health in football is to conduct comprehensive testing prior to commitment to a professional league or tournament. This testing can include specific biological measures, such as genetic testing, biomechanical analysis, or hormonal testing. Identifying predisposing factors that increase injury risk for women would enable the creation of tailored IPPs, ensuring more effective and targeted measures.

In conclusion, the increasing participation of women in football underscores the urgent need for research and implementation of injury prevention strategies tailored specifically to female athletes. The heightened risk of injuries, such as ACL ruptures, driven by biological, physiological, and hormonal factors unique to women, demands targeted approaches in IPPs. Current programs like FIFA 11+ show promise but require adaptation to address the specific needs of female football players effectively. Additionally, the mental health challenges faced by female athletes further emphasize the necessity of a holistic approach to their well-being. Addressing these gaps through comprehensive, sex-specific interventions could significantly reduce injury rates, improve long-term health outcomes, and enhance the overall athletic experience for women in football.

## Data Availability

No data were created during the writing of this manuscript.

## References

[1] Williams J., Hess R. (2015). Women, Football and History: International Perspectives. Int. J. Hist. Sport.

[2] Zeen K. A Brief History of Women’s Football. https://www.sundried.com/blogs/training/a-brief-history-of-womens-football?currency=GBP.

[3] Mandelbaum B., D’Hooghe P. (2023). Female Athlete Health in Women’s Football. Aspetar Sports Med. J..

[4] Podewils K. (2023). The Continuing Rise of Women’s Sport.

[5] Fahmy M. Increased participation and competitions. Proceedings of the 5th FIFA Women’s Football Symposium.

[6] FIFA (2023). Women’s Football: Member Associations Survey Report. https://digitalhub.fifa.com/m/28ed34bd888832a8/original/FIFA-Women-s-Football-MA-Survey-Report-2023.pdf.

[7] Le Gall F., Carling C., Reilly T. (2008). Injuries in young elite female soccer players. Am. J. Sports Med..

[8] Martín-San Agustín R., Medina-Mirapeix F., Esteban-Catalán A., Escriche-Escuder A., Sánchez-Barbadora M., Benítez-Martínez J.C. (2021). Epidemiology of Injuries in First Division Spanish Women’s Soccer Players. Int. J. Environ. Res. Public Health.

[9] Clausen M.B., Zebis M.K., Møller M., Krustrup P., Hölmich P., Wedderkopp N., Andersen L.L., Christensen K.B., Thorborg K. (2014). High Injury Incidence in Adolescent Female Soccer. Am. J. Sports Med..

[10] Grygorowicz M., Michałowska M., Jurga P., Piontek T., Jakubowska H., Kotwicki T. (2019). Thirty Percent of Female Footballers Terminate Their Careers Due to Injury: A Retrospective Study Among Polish Former Players. J. Sport Rehabil..

[11] Mendonça L.D., Ley C., Schuermans J., Wezenbeek E., IFSPT, Witvrouw E. (2021). How Injury Prevention Programs Are Being Structured and Implemented Worldwide: An International Survey of Sports Physical Therapists. Phys. Ther. Sport.

[12] Lindenfeld T.N., Schmitt D.J., Hendy M.P., Mangine R.E., Noyes F.R. (1994). Incidence of injury in indoor soccer. Am. J. Sports Med..

[13] Arendt E.A., Agel J., Dick R. (1999). Anterior cruciate ligament injury patterns among collegiate men and women. J. Athl. Train..

[14] Emery C.A., Roy T.-O., Whittaker J.L., Nettel-Aguirre A., van Mechelen W. (2015). Neuromuscular training injury prevention strategies in youth sport: A systematic review and meta-analysis. Br. J. Sports Med..

[15] Lin C.Y., Casey E., Herman D.C., Katz N., Tenforde A.S. (2018). Sex differences in common sports injuries. PM&R.

[16] Arundale A.J.H., Silvers-Granelli H.J., Myklebust G. (2022). ACL Injury Prevention: Where Have We Come from and Where Are We Going?. J. Orthop. Res..

[17] Montalvo A.M., Schneider D.K., Silva P.L., Yut L., Webster K.E., Riley M.A., Kiefer A.W., Doherty-Restrepo J.L., Myer G.D. (2018). What’s My Risk of Sustaining an ACL Injury While Playing Football (Soccer)? A Systematic Review with Meta-Analysis. Br. J. Sports Med..

[18] Crossley K.M., Patterson B.E., Culvenor A.G., Bruder A.M., Mosler A.B., Mentiplay B.F. (2020). Making football safer for women: A systematic review and meta-analysis of injury prevention programmes in 11,773 female football (soccer) players. Br. J. Sports Med..

[19] Al Attar W.S.A., Alshehri M.A. (2019). A meta-analysis of meta-analyses of the effectiveness of FIFA injury prevention programs in soccer. Scand. J. Med. Sci. Sports.

[20] Michaelidis M., Koumantakis G.A. (2014). Effects of Knee Injury Primary Prevention Programs on Anterior Cruciate Ligament Injury Rates in Female Athletes in Different Sports: A Systematic Review. Phys. Ther. Sport.

[21] Åman M., Larsén K., Forssblad M., Näsmark A., Waldén M., Hägglund M. (2018). A Nationwide Follow-up Survey on the Effectiveness of an Implemented Neuromuscular Training Program to Reduce Acute Knee Injuries in Soccer Players. Orthop. J. Sports Med..

[22] Iván-Baragaño I., Maneiro R., Losada J.L., Ardá A. (2022). Influence of match status in ball possessions in the FIFA Women’s World Cup France 2019. Proc. Inst. Mech. Eng. P J. Sports Eng. Technol..

[23] Foulis M. (2022). Women three times more likely to get injured than men. Safety Magazine.

[24] Sallis R.E., Jones K., Sunshine S., Smith G., Simon L. (2001). Comparing Sports Injuries in Men and Women. Int. J. Sports Med..

[25] Hallén A., Tomás R., Ekstrand J., Bengtsson H., den Steen E.V., Hägglund M., Waldén M. (2024). UEFA Women’s Elite Club Injury Study: A prospective study on 1527 injuries over four consecutive seasons 2018/2019 to 2021/2022 reveals thigh muscle injuries to be most common and ACL injuries most burdensome. Br. J. Sports Med..

[26] Mayo Clinic ACL Injury—Symptoms and Causes. https://www.mayoclinic.org/diseases-conditions/acl-injury/symptoms-causes/syc-20350738.

[27] Alentorn-Geli E., Myer G.D., Silvers H.J., Samitier G., Romero D., Lázaro-Haro C., Cugat R. (2009). Prevention of non-contact anterior cruciate ligament injuries in soccer players. Part 1: Mechanisms of injury and underlying risk factors. Knee Surg. Sports Traumatol. Arthrosc..

[28] Ireland M.L. (2002). The female ACL: Why is it more prone to injury?. Orthop. Clin. N. Am..

[29] Waldén M., Hägglund M., Werner J., Ekstrand J. (2011). The Epidemiology of Anterior Cruciate Ligament Injury in Football (Soccer): A Review of the Literature from a Gender-Related Perspective. Knee Surg. Sports Traumatol. Arthrosc..

[30] Shultz S.J., Sander T.C., Kirk S.E., Perrin D.H. (2005). Sex Differences in Knee Joint Laxity Change Across the Female Menstrual Cycle. J. Sports Med. Phys. Fit..

[31] Martin D., Timmins K., Cowie C. (2021). Injury Incidence Across the Menstrual Cycle in International Footballers. Front. Sports Act. Living.

[32] Smith R. Female Athletes Six Times More Likely to Get Injured in the Days Leading Up to Their Period. https://www.technologynetworks.com/proteomics/news/female-athletes-six-times-more-likely-to-get-injured-in-the-days-leading-up-to-their-period-386458.

[33] Wojtys E.M., Huston L.J., Lindenfeld T.N., Hewett T.E., Greenfield M.L.V.H. (1998). Association Between the Menstrual Cycle and Anterior Cruciate Ligament Injuries in Female Athletes. Am. J. Sports Med..

[34] Wojtys E.M., Huston L.J., Boynton M.D., Spindler K.P., Lindenfeld T.N. (2002). The Effect of the Menstrual Cycle on Anterior Cruciate Ligament Injuries in Women as Determined by Hormone Levels. Am. J. Sports Med..

[35] Wild C.Y., Steele J.R., Munro B.J. (2012). Why Do Girls Sustain More Anterior Cruciate Ligament Injuries Than Boys?. Sports Med..

[36] Myer G.D., Ford K.R., Paterno M.V., Nick T.G., Hewett T.E. (2008). The Effects of Generalized Joint Laxity on Risk of Anterior Cruciate Ligament Injury in Young Female Athletes. Am. J. Sports Med..

[37] Ahmetov I.I., Stepanova A.A., Biktagirova E.M., Semenova E.A., Shchuplova I.S., Bets L.V., Andryushchenko L.B., Borisov O.V., Andryushchenko O.N., Generozov E.V. (2020). Is testosterone responsible for athletic success in female athletes?. J. Sports Med. Phys. Fit..

[38] McFadden B.A., Walker A.J., Bozzini B.N., Hofacker M., Russell M., Arent S.M. (2022). Psychological and Physiological Changes in Response to the Cumulative Demands of a Women's Division I Collegiate Soccer Season. J. Strength Cond. Res..

[39] Lovering R.M., Romani W.A. (2005). Effect of Testosterone on the Female Anterior Cruciate Ligament. Am. J. Physiol. Regul. Integr. Comp. Physiol..

[40] Oberoi P. (2024). Women Athletes Deserve Better Health Resources. Forbes. https://www.forbes.com/sites/priyaoberoi/2024/06/13/women-athletes-deserve-better-health-resources/.

[41] Russell K.A., Palmieri R.M., Zinder S.M., Ingersoll C.D. (2006). Sex Differences in Valgus Knee Angle During a Single-Leg Drop Jump. J. Athl. Train..

[42] Cheung E.C., Boguszewski D.V., Joshi N.B., Wang D., McAllister D.R. (2015). Anatomic Factors that May Predispose Female Athletes to Anterior Cruciate Ligament Injury. Curr. Sports Med. Rep..

[43] Hashemi J., Mansouri H., Chandrashekar N., Slauterbeck J.R., Hardy D.M., Beynnon B.D. (2011). Age, sex, body anthropometry, and ACL size predict the structural properties of the human anterior cruciate ligament. J. Orthop. Res..

[44] Hashemi J., Chandrashekar N., Gill B., Beynnon B.D., Slauterbeck J.R., Schutt R.C., Mansouri H., Dabezies E. (2008). The geometry of the tibial plateau and its influence on the biomechanics of the tibiofemoral joint. J. Bone Jt. Surg. Am..

[45] Lipps D.B., Oh Y.K., Ashton-Miller J.A., Wojtys E.M. (2011). Morphologic characteristics help explain the gender difference in peak anterior cruciate ligament strain during a simulated pivot landing. Am. J. Sports Med..

[46] LaPrade R.F., Burnett Q.M. (1994). Femoral intercondylar notch stenosis and correlation to anterior cruciate ligament injuries. Am. J. Sports Med..

[47] Whitney D.C., Sturnick D.R., Vacek P.M., DeSarno M.J., Gardner-Morse M., Tourville T.W., Smith H.C., Slauterbeck J.R., Johnson R.J., Shultz S.J. (2014). Relationship Between the Risk of Suffering a First-Time Noncontact ACL Injury and Geometry of the Femoral Notch and ACL. Am. J. Sports Med..

[48] Garvey K.D., Lowenstein N.A., Matzkin E.G., Frank R.M. (2022). Chapter 5—Anterior Cruciate Ligament Injury Prevention. The Female Athlete.

[49] Huston L.J., Wojtys E.M. (1996). Neuromuscular performance characteristics in elite female athletes. Am. J. Sports Med..

[50] Wojtys E.M., Huston L.J., Taylor P.D., Bastian S.D. (1996). Neuromuscular Adaptations in Isokinetic, Isotonic, and Agility Training Programs. Am. J. Sports Med..

[51] Hewett T.E., Myer G.D., Ford K.R., Heidt R.S., Colosimo A.J., McLean S.G., van den Bogert A.J., Paterno M.V., Succop P. (2005). Biomechanical measures of neuromuscular control and valgus loading of the knee predict anterior cruciate ligament injury risk in female athletes: A prospective study. Am. J. Sports Med..

[52] Zebis M.K., Aagaard P., Andersen L.L., Hölmich P., Clausen M.B., Brandt M., Husted R.S., Lauridsen H.B., Curtis D.J., Bencke J. (2022). First-time anterior cruciate ligament injury in adolescent female elite athletes: A prospective cohort study to identify modifiable risk factors. Knee Surg. Sports Traumatol. Arthrosc..

[53] Ireland M.L., Bolgla L.A., Noehren B., Noyes F.R., Barber-Westin S. (2018). Gender differences in core strength and lower extremity function during static and dynamic single-leg squat tests. ACL Injuries in the Female Athlete.

[54] De Blaiser C., De Ridder R., Willems T., Vanden Bossche L., Danneels L., Roosen P. (2019). Impaired Core Stability as a Risk Factor for the Development of Lower Extremity Overuse Injuries: A Prospective Cohort Study. Am. J. Sports Med..

[55] Cresswell A.G., Grundström H., Thorstensson A. (1992). Observations on intra-abdominal pressure and patterns of abdominal intra-muscular activity in man. Acta Physiol. Scand..

[56] Richardson C., Toppenberg R., Jull G. (1990). An Initial Evaluation of Eight Abdominal Exercises for Their Ability to Provide Stabilisation for the Lumbar Spine. Aust. J. Physiother..

[57] De Blaiser C., Roosen P., Willems T., Danneels L., Bossche L.V., De Ridder R. (2018). Is core stability a risk factor for lower extremity injuries in an athletic population? A systematic review. Phys. Ther. Sport.

[58] Leetun D.T., Ireland M.L., Willson J.D., Ballantyne B.T., Davis I.M. (2004). Core stability measures as risk factors for lower extremity injury in athletes. Med. Sci. Sports Exerc..

[59] Greene F.S., Perryman E., Cleary C.J., Cook S.B. (2019). Core Stability and Athletic Performance in Male and Female Lacrosse Players. Int. J. Exerc. Sci..

[60] Gordon A.T., Ambegaonkar J.P., Caswell S.V. (2013). Relationships between core strength, hip external rotator muscle strength, and star excursion balance test performance in female lacrosse players. Int. J. Sports Phys. Ther..

[61] Zazulak B.T., Hewett T.E., Reeves N.P., Goldberg B., Cholewicki J. (2007). The Effects of Core Proprioception on Knee Injury. Am. J. Sports Med..

[62] Gouttebarge V., Frings-Dresen M.H., Sluiter J.K. (2015). Mental and Psychosocial Health among Current and Former Professional Footballers. Occup. Med..

[63] Bilgoe S.C., Goedhart E., Orhant E., Kerkhoffs G., Gouttebarge V. (2024). Unmasking Mental Health Symptoms in Female Professional Football Players: A 12-Month Follow-Up Study. BMJ Open Sport Exerc. Med..

[64] Muuns A. (2021). Mental Health and Performance in Professional Female Football Players. LinkedIn Pulse. https://www.linkedin.com/pulse/mental-health-performance-professional-female-football-munns.

[65] Junge A., Prinz B. (2019). Depression and Anxiety Symptoms in 17 Teams of Female Football Players Including 10 German First League Teams. Br. J. Sports Med..

[66] Salam R.A., Arshad A., Das J.K., Khan M.N., Mahmood W., Freedman S.B., Bhutta Z.A. (2016). Interventions to Prevent Unintentional Injuries Among Adolescents: A Systematic Review and Meta-Analysis. J. Adolesc. Health.

[67] Al Attar W., Soomro N., Sinclair P., Pappas E., Sanders R. (2015). How effective are F-MARC injury prevention programs for soccer players? A systematic review and meta-analysis. J. Sci. Med. Sport.

[68] Al Attar W.S.A., Majrashi A., Bizzini M. (2024). Effectiveness of FIFA 11+ Injury Prevention Programs in Reducing Head and Neck Injuries, Including Concussion, Among Soccer Players: A Systematic Review and Meta-Analysis. Pediatr. Exerc. Sci..

[69] Soligard T., Myklebust G., Steffen K., Holme I., Silvers H., Bizzini M., Junge A., Dvorak J., Bahr R., Andersen T.E. (2008). Comprehensive Warm-Up Programme to Prevent Injuries in Young Female Footballers: Cluster Randomised Controlled Trial. BMJ.

[70] Ling D.I., Cepeda N.A., Marom N., Jivanelli B., Marx R.G. (2020). Injury prevention programmes with plyometric and strengthening exercises improve on-field performance: A systematic review. J. ISAKOS.

[71] Ramos A.P., de Mesquita R.S., Migliorini F., Maffulli N., Okubo R. (2024). FIFA 11+ Kids in the Prevention of Soccer Injuries in Children: A Systematic Review. J. Orthop. Surg. Res..

[72] Rössler R., Verhagen E., Rommers N., Dvorak J., Junge A., Lichtenstein E., Donath L., Faude O. (2019). Comparison of the ‘11+ Kids’ Injury Prevention Programme and a Regular Warm-Up in Children’s Football (Soccer): A Cost Effectiveness Analysis. Br. J. Sports Med..

[73] Althomali O.W., Ibrahim A.A., Algharbi A.F., Alshammari S.S., Alajlan S.N., Albaqawi J.A., Alshammari A.F., Sheeha B.B., Hussein H.M. (2025). The FIFA 11+ Injury Prevention Program Reduces the Incidence of Lower Extremity Injuries in Football Players: A Systematic Review and Meta-Analysis. J. Sports Med. Phys. Fit..

[74] Scott C. A Training Program to Prevent Leg Injuries in Community Australian Football. https://coastsport.com.au/wp-content/uploads/2018/02/FootyFirst_-_Manual.pdf.

[75] Bailey M. (2023). How to Tackle the Increased Rate of ACL Injuries in Women’s Football. BOA Website. https://www.boa.ac.uk/resource/how-to-tackle-the-increased-rate-of-acl-injuries-in-women-s-football.html.

[76] Magnusson K., Turkiewicz A., Hughes V., Frobell R., Englund M. (2020). High Genetic Contribution to Anterior Cruciate Ligament Rupture: Heritability ~69. Br. J. Sports Med..

[77] Juffer P., Furrer R., González-Freire M., Santiago C., Verde Z., Serratosa L., Morate F.J., Rubio J.C., Martin M.A., Ruiz J.R. (2009). Genotype distributions in top-level soccer players: A role for ACE?. Int. J. Sports Med..

[78] Pickering C., Suraci B., Semenova E.A., Boulygina E.A., Kostryukova E.S., Kulemin N.A., Borisov O.V., Khabibova S.A., Larin A.K., Pavlenko A.V. (2019). A genome-wide association study of sprint performance in elite youth football players. J. Strength Cond. Res..

[79] Murtagh C.F., Brownlee T.E., Rienzi E., Roquero S., Moreno S., Huertas G., Lugioratto G., Baumert P., Turner D.C., Lee D. (2020). The genetic profile of elite youth soccer players and its association with power and speed depends on maturity status. PLoS ONE.

[80] Pruna R., Artells R., Ribas J., Montoro B., Cos F., Muñoz C., Rodas G., Maffulli N. (2013). Single nucleotide polymorphisms associated with non-contact soft tissue injuries in elite professional soccer players: Influence on degree of injury and recovery time. BMC Musculoskelet. Disord..

[81] Pruna R., Artells R., Lundblad M., Maffulli N. (2017). Genetic biomarkers in non-contact muscle injuries in elite soccer players. Knee Surg. Sports Traumatol. Arthrosc..

[82] Larruskain J., Celorrio D., Barrio I., Odriozola A., Gil S.M., Fernandez-Lopez J.R., Nozal R., Ortuzar I., Lekue J.A., Aznar J.M. (2018). Genetic Variants and Hamstring Injury in Soccer: An Association and Validation Study. Med. Sci. Sports Exerc..

[83] Hall E.C.R., Baumert P., Larruskain J., Gil S.M., Lekue J.A., Rienzi E., Moreno S., Tannure M., Murtagh C.F., Ade J.D. (2022). The genetic association with injury risk in male academy soccer players depends on maturity status. Scand. J. Med. Sci. Sports.

[84] Ficek K., Cieszczyk P., Kaczmarczyk M., Maciejewska-Karłowska A., Sawczuk M., Cholewinski J., Leonska-Duniec A., Stepien-Slodkowska M., Zarebska A., Stepto N.K. (2013). Gene variants within the COL1A1 gene are associated with reduced anterior cruciate ligament injury in professional soccer players. J. Sci. Med. Sport.

[85] Massidda M., Bachis V., Corrias L., Piras F., Scorcu M., Calò C.M. (2015). Influence of the COL5A1 rs12722 on musculoskeletal injuries in professional soccer players. J. Sports Med. Phys. Fit..

[86] Clos E., Pruna R., Lundblad M., Artells R., Esquirol Caussa J. (2019). ACTN3 single nucleotide polymorphism is associated with non-contact musculoskeletal soft-tissue injury incidence in elite professional football players. Knee Surg. Sports Traumatol. Arthrosc..

[87] Massidda M., Voisin S., Culigioni C., Piras F., Cugia P., Yan X., Eynon N., Calò C.M. (2019). ACTN3 R577X Polymorphism Is Associated With the Incidence and Severity of Injuries in Professional Football Players. Clin. J. Sport Med..

[88] Rodas G., Moreno-Pérez V., Del Coso J., Florit D., Osaba L., Lucia A. (2021). Alpha-Actinin-3 Deficiency Might Affect Recovery from Non-Contact Muscle Injuries: Preliminary Findings in a Top-Level Soccer Team. Genes.

[89] Gabbasov R.T., Arkhipova A.A., Borisova A.V., Hakimullina A.M., Kuznetsova A.V., Williams A.G., Day S.H., Ahmetov I.I. (2013). The HIF1A Gene Pro582Ser Polymorphism in Russian Strength Athletes. J. Strength Cond. Res..

[90] Maciejewska-Skrendo A., Sawczuk M., Cięszczyk P., Ahmetov I.I., Barh D., Ahmetov I. (2019). Genes and power athlete status. Sports, Exercise, and Nutritional Genomics: Current Status and Future Directions.

[91] Moreland E., Borisov O.V., Semenova E.A., Larin A.K., Andryushchenko O.N., Andryushchenko L.B., Generozov E.V., Williams A.G., Ahmetov I.I. (2022). Polygenic Profile of Elite Strength Athletes. J. Strength Cond. Res..

[92] Murtagh C.F., Hall E.C.R., Brownlee T.E., Drust B., Williams A.G., Erskine R.M. (2023). The Genetic Association with Athlete Status, Physical Performance, and Injury Risk in Soccer. Int. J. Sports Med..

[93] Gineviciene V., Utkus A., Pranckevičienė E., Semenova E.A., Hall E.C.R., Ahmetov I.I. (2022). Perspectives in Sports Genomics. Biomedicines.

[94] Eynon N., Nasibulina E.S., Banting L.K., Cieszczyk P., Maciejewska-Karlowska A., Sawczuk M., Bondareva E.A., Shagimardanova R.R., Raz M., Sharon Y. (2013). The FTO A/T polymorphism and elite athletic performance: A study involving three groups of European athletes. PLoS ONE.

[95] Semenova E.A., Miyamoto-Mikami E., Akimov E.B., Al-Khelaifi F., Murakami H., Zempo H., Kostryukova E.S., Kulemin N.A., Larin A.K., Borisov O.V. (2020). The association of HFE gene H63D polymorphism with endurance athlete status and aerobic capacity: Novel findings and a meta-analysis. Eur. J. Appl. Physiol..

[96] Wang G., Tanaka M., Eynon N., North K.N., Williams A.G., Collins M., Moran C.N., Britton S.L., Fuku N., Ashley E.A. (2016). The Future of Genomic Research in Athletic Performance and Adaptation to Training. Genet. Sports.

[97] Boulygina E.A., Borisov O.V., Valeeva E.V., Semenova E.A., Kostryukova E.S., Kulemin N.A., Larin A.K., Nabiullina R.M., Mavliev F.A., Akhatov A.M. (2020). Whole genome sequencing of elite athletes. Biol. Sport.

[98] Ahmetov I.I., John G., Semenova E.A., Hall E.C.R. (2024). Genomic predictors of physical activity and athletic performance. Adv. Genet..

[99] Hall E.C.R., John G., Ahmetov I.I. (2024). Testing in Football: A Narrative Review. Sports.

[100] Akhmetov I.I., Astranenkova I.V., Rogozkin V.A. (2007). Association of PPARD gene polymorphism with human physical performance. Mol. Biol..

[101] Domańska-Senderowska D., Snochowska A., Szmigielska P., Jastrzębski Z., Jegier A., Kiszałkiewicz J., Dróbka K., Jastrzębska J., Pastuszak-Lewandoska D., Cięszczyk P. (2018). Analysis of the PPARD Gene Expression Level Changes in Football Players in Response to the Training Cycle. Balkan J. Med. Genet..

[102] Kaplan T.A., Digel S.L., Scavo V.A., Arellana S.B. (1995). Effect of Obesity on Injury Risk in High School Football Players. Clin. J. Sport Med..

[103] Dvorak J., Junge A., Chomiak J., Graf-Baumann T., Peterson L., Rösch D., Hodgson R. (2000). Risk Factor Analysis for Injuries in Football Players: Possibilities for a Prevention Program. Am. J. Sports Med..

[104] López-Valenciano A., Raya-González J., Garcia-Gómez J.A., Aparicio-Sarmiento A., Sainz de Baranda P., De Ste Croix M., Ayala F. (2021). Injury Profile in Women’s Football: A Systematic Review and Meta-Analysis. Sports Med..

[105] Zech A., Hollander K., Junge A., Steib S., Groll A., Heiner J., Nowak F., Pfeiffer D., Rahlf A.L. (2022). Sex differences in injury rates in team-sport athletes: A systematic review and meta-regression analysis. J. Sport Health Sci..

